# Epigenetic Modulating Agents as a New Therapeutic Approach in Multiple Myeloma

**DOI:** 10.3390/cancers5020430

**Published:** 2013-04-15

**Authors:** Ken Maes, Eline Menu, Els Van Valckenborgh, Ivan Van Riet, Karin Vanderkerken, Elke De Bruyne

**Affiliations:** 1 Department of Hematology and Immunology, Myeloma Center Brussels, Vrije Universiteit Brussel (VUB), Laarbeeklaan 103, 1090 Brussel, Belgium; 2 Stem Cell Laboratory, Department Clinical Hematology, Universitair Ziekenhuis Brussel (UZ Brussel), Laarbeeklaan 101, 1090 Brussel, Belgium

**Keywords:** multiple myeloma, epigenetics, histone deacetylase inhibitor, DNA-methyltransferase inhibitor

## Abstract

Multiple myeloma (MM) is an incurable B-cell malignancy. Therefore, new targets and drugs are urgently needed to improve patient outcome. Epigenetic aberrations play a crucial role in development and progression in cancer, including MM. To target these aberrations, epigenetic modulating agents, such as DNA methyltransferase inhibitors (DNMTi) and histone deacetylase inhibitors (HDACi), are under intense investigation in solid and hematological cancers. A clinical benefit of the use of these agents as single agents and in combination regimens has been suggested based on numerous studies in pre-clinical tumor models, including MM models. The mechanisms of action are not yet fully understood but appear to involve a combination of true epigenetic changes and cytotoxic actions. In addition, the interactions with the BM niche are also affected by epigenetic modulating agents that will further determine the *in vivo* efficacy and thus patient outcome. A better understanding of the molecular events underlying the anti-tumor activity of the epigenetic drugs will lead to more rational drug combinations. This review focuses on the involvement of epigenetic changes in MM pathogenesis and how the use of DNMTi and HDACi affect the myeloma tumor itself and its interactions with the microenvironment.

## 1. Introduction

Multiple myeloma (MM) is a clonal B-cell malignancy characterized by the uncontrolled growth of malignant plasma cells in the bone marrow, resulting in an accumulation of monoclonal immunoglobulins (M-spike) in the blood and urine of the patient. Common symptoms at diagnosis include bone lesions, hypercalcemia, kidney failure, anemia and recurrent infections [1,2]. The annual incidence rate in most developed countries is approximately five cases per 100,000 [3,4]. Of outmost importance for the growth of the MM tumor is the interaction with the bone marrow (BM) microenvironment. There, various bidirectional interactions between MM cells and the BM compartment stimulate survival, growth, drug resistance, migration and immune escape of MM cells. In addition, increased angiogenesis and bone lesions further contribute to MM development [1]. The above interactions are mediated by secretion of multiple cytokines, including interleukin-6 (IL-6), insulin-like growth factor-1 (IGF-1), IL-1, tumor necrosis factor-α (TNF-α), vascular endothelial growth factor (VEGF), Dickkopf-related protein 1 (DKK1) and secreted frizzled-related protein (sFRP), that support paracrine interactions between MM cells and endothelial cells, bone marrow stromal cells (BMSC), osteoblasts, osteoclasts and immune cells. In addition, direct cell-cell and cell-matrix interactions by expression of adhesion molecules such as integrin-α4β1 (VLA-4) and CD138 (syndecan-1) on MM cells stimulate the expression and secretion of growth factors into the BM microenvironment [1,5]. 

MM tumors evolve from a pre-malignant state called “monoclonal gammopathy of undetermined significance” or MGUS. MGUS evolves to symptomatic MM with a rate of 1% per year [6,7]. In advanced cases, MM cells migrate to extramedullary sites like liver and spleen what can be associated with primary or secondary plasma cell leukemia (PCL) [8]. The progression from MGUS to MM and PCL involves accumulating cytogenetic abnormalities. The initial or primary aberrations involved in MGUS and MM development can be divided in two subgroups: (I) hyperdiploidy (multiple trisomies) and (II) translocations of the immunoglobulin-G heavy chain locus on chromosome 14. Different translocation partners have been described such as t(4;14), t(11;14) and t(14;16 or 20), which respectively affect the following genes: *FGFR3/MMSET*, *CCND* (*cyclin D* family) and *MAF* genes [8,9]. Upregulation of the *CCND* family is a common event in most MM tumors. Progression of MGUS to MM and PCL is associated with additional aberrations including activating *RAS* mutations, deletion of 13q or 17p, *MYC* overexpression and mutations of *TP53* [8,9].

The treatment options for newly diagnosed patients are based on transplantation eligibility (relies on age and co-morbidities) and risk stratification. In general, high risk-patients are characterized by t(14;16), t(14;20), t(4;14), 17p or 13q deletion while all other patients have a standard-risk [10,11]. However, this stratification is not always clear cut. For example, some classify t(4;14) as an intermediate risk if it is not associated with a high risk-gene expression profile [12], whereas Avet-Loiseau *et al*. demonstrated the lack of prognostic value of t(14;16) [13]. Patients are currently treated according to the risk with different combinations of (novel) agents such as proteasome inhibitors (bortezomib), immunomodulatory drugs (thalidomide, lenalidomide), alkylators (cyclophosphamide, melphalan) or glucocorticoids (dexamethasone, prednisone) followed by an autologous transplantion if eligible. Transplantation is then mostly followed by a bortezomib- or lenalidomide-based maintenance therapy. The introduction of these novel agents and regimens led to an overall survival of 5–7 years in standard-risk patients [14,15,16]. Despite the promising clinical effects of the current therapies, patients eventually relapse and die of refractory disease. This emphasizes the need for novel targets and treatment options. 

Currently, there is much interest in the use of epigenetic modulating agents as an alternative approach for cancer therapy. This review will focus on recent knowledge of epigenetic aberrations in MM and the use of epigenetic modulating agents, in particular histone deacetylase inhibitors (HDACi) and DNA-methyltransferase inhibitors (DNMTi) in MM.

## 2. Epigenetics

Epigenetics is defined as the study of all heritable changes in gene expression that occur independent of changes in the primary DNA sequence [17]. Most of the epigenetic mechanisms occur at the level of chromatin, the higher order structure of DNA. Chromatin is built up by nucleosomes which contain ±146 bp of DNA wrapped around an octamer of four core histones (H2A, H2B, H3 and H4). The chromatin structure and compactness is mostly defined by epigenetic mechanisms including DNA methylation, post-translational modifications of histones and nucleosome positioning. The dynamics of these modifications will define the accessibility of the transcriptional machinery towards chromatin regions and thus gene expression. In addition, microRNAs form an additional layer of post-transcriptional control by affecting mRNA levels of genes [17].

### 2.1. DNA Methylation

DNA methylation is the covalent addition of a methyl-molecule on the 5' cytosine residue (5mC) preceding a guanine residue in so called CpG dinucleotides. This reaction is catalyzed by DNA methyltransferases (DNMTs) and uses S-adenosyl-L-methionine (SAM) as a methyl donor [18]. DNA methylation patterns are acquired *de novo* during early development and lineage commitment and are established by DNMT3a and DNMT3b. Maintenance of DNA patterns upon cell division is then executed by DNMT1 and governs heritability of methylation patterns. However, evidence shows now that there is a large redundancy present in the functions of the different DNMTs [19,20]. In the mammalian genome, CpG dinucleotides can be found in long repetitive stretches (such as centromers and telomeres) where they are highly methylated and this maintains genomic stability [21]. In addition, CpG dinucleotides are enriched in CpG islands (CGIs) located at the 5' flanking promoter regions of genes, close to their transcriptional start site (TSS). It is estimated that 50–60% of gene promoters contain CGIs [22]. These CGIs are normally unmethylated and permissive for transcription. However, a small subset of the CGIs is methylated, leading to permanent gene silencing. This is for example the case for tissue specific genes, germline specific genes, imprinted genes and X-chromosome inactivation in females [23,24]. CGI poor-promoters are also subjected to DNA methylation close to their TSS and like in CGI-rich promoters; this negatively correlates with gene expression. However, CpG sites are also found within gene bodies and methylation of these sites positively correlates with gene expression [25]. Nonetheless, the function of gene body methylation remains to be identified. In summary, the outcome of DNA methylation is dependent on the location within the gene. The process of gene silencing is not only dependent on DNA methylation but involves other epigenetic modifications as well, such as histone modification and chromatin remodeling. 5mC can be recognized by proteins containing methyl binding domains (MBD). In that way, proteins that mediate repressive histone modifications and chromatin remodeling are recruited. It is the cross-talk between these proteins that contribute to (stable) gene silencing [24]. Only recently, several mechanisms for DNA demethylation have been proposed. DNA demethylation is possible through enzymatic activity and includes conversion of 5mC by deamination to thymine (catalyzed by AID) or by hydroxylation to hydroxyl-methyl cytosine (5hmC; catalyzed by the TET family). Subsequently, DNA repair mechanisms such as base-excision repair and nucleotide-excision repair are initiated that remove the modified cytosine [19].

### 2.2. Histone Modifications

The *N*-terminal tails of histones are subjected to a plethora of post-translational modifications, including acetylation, methylation, phosphorylation, ubiquitination, sumoylation and glycosylation. Histone modifications are the result of a complex interplay between different molecules referred to as chromatin modifying proteins subdivided in “writers”, “readers” and “erasers” [26]. Writers are enzymes that catalyze the actual modification while readers are proteins that contain certain domains that recognize the different types of modifications. Methylated residues can be recognized by PHD fingers and the Tudor “royal” family containing tudor domains, chromodomains and MBT domains. Acetylated residues are recognized by bromodomains and phosphorylated residues by a domain in 14-3-3 proteins. Erasers consist of enzymes that remove histone modifications. Thus, histone modifications recruit, in a context dependent manner, specific protein complexes consisting of different writers, erasers and chromatin accessory proteins. Thereby, they orchestrate various functions related to chromatin including transcription, DNA repair, chromatin structure and DNA replication [26,27]. The most studied modifications that play a key role in chromatin biology are acetylation, methylation and phosphorylation and are introduced below.

#### 2.2.1. Histone Acetylation

In general, acetylation of lysine (K) residues of histone tails affects the interaction of histone tails and DNA in nucleosomes by means of neutralizing the negative electric charge of DNA. Hyperacetylation results in a more relaxed state of chromatin and enhances accessibility of the transcription machinery and other chromatin accessory proteins. In contrast, hypoacetylation leads to a more compact chromatin state and is involved in gene silencing. The balance of histone acetylation depends on the activity of two enzyme groups: histone acetyltransferases (HATs, “writers”) and histone deacetylases (HDACs, “erasers”) [28]. At least two different classes of HATs exist in mammalians: type-A and type-B. Type-A HATs consist of three families: GNAT, MYST and p300/CBP. These HATs are part of multiprotein complexes in the nucleus and acetylate *N*-terminal tails of histones within the chromatin. Type-B HATs (HAT1 and HAT2) are located in the cytoplasm and acetylate newly synthesized histones before assembly [28,29]. HDAC proteins can be divided into four classes: Class I HDACs (HDAC-1, -2, -3 and -8) are exclusively found in the nucleus. Class II (HDAC-4, -5, -6, -7, -9 and -10) are able to shuttle between nucleus and cytoplasm and contain two deacetylase domains. HDAC 11 represents class IV because of the low sequence similarity with other HDACs. Class I, II and IV HDACs all require Zn+ for their catalytic activity. In contrast, class III HDACs (also called sirtuins) are dependent on NAD+ for their catalytic activity [30]. Histone tails contain several lysine residues that have been described to be acetylated. Acetylation sites linked with transcriptional activation are for example histone-3-lysine-9 acetylation (H3K9ac), H3K14ac, H3K18ac and H4K5ac. Other functions are also mediated by histone acetylation such as DNA repair (H4K8ac, H3K56ac) and chromatin remodeling (H4K16ac, H2BK12ac) [28]. Acetylation is not solely reserved for histones but also various other types of proteins are subjected to acetylation. Mass spectrometry revealed acetylation of proteins involved in transcription, translation, splicing, DNA repair, cell cycle progression, protein folding, cytoskeleton dynamics, signal transduction and metabolism [31]. The list of proteins that are known to be acetylated is expanding and include p53, HSP90, tubulin, NF-κB, HIF-1α, RUNX3, STAT-3, E2F1, Ku70 and c-MYC. Acetylation thus functions as a broad post-translational modification regulating protein functions including DNA-binding, activity of transcription factors, subcellular localization and protein stability [31,32].

#### 2.2.2. Histone Methylation

Histone methylation represents an important histone modification that is involved in both activation and repression of transcription. Both lysine and arginine (R) residues in histone tails are subjected to methylation. The fact that these residues can be mono-, di-, or tri-methylated (only K) underlies the complexity and mediates the differential functions of histone methylation [20]. The enzymes responsible for histone methylation are grouped in lysine and arginine histone methyltransferases (KHMT and PRMT respectively; “writers”) and use SAM as methyl donor. Various human KHMTs have been described including SETD1, SUV39H1, G9a, EZH2, MLL1-5 and NSD1-3. The common SET domain in KHMTs is responsible for the enzymatic activity. Lysine methylation sites that are linked with transcriptional activation are di-, tri-methylation of H3K4 (H3K4me2/3), H3K36me2/3 and H3K79me. Repressive marks include H3K9me2/3, H3K27me2/3 and H4K20me3 [20,29]. The family of PRMTs consists of PRMT1-9, CARM1 and FBXO11. Arginine residues on both H3 and H4 can be methylated and include H3R2, H3R17, H3R26 and H4R3. At the functional level, arginine methylation can regulate transcriptional activation and repression, mRNA splicing, DNA repair and signal transduction [33]. Histone methylation is also a reversible modification as evidenced by the discovery of histone demethylases (HDM). Human examples include lysine specific demethylases (KMD1-5, also known as LSD-1) and JumonjiC (JmjC)-domain containing demethylases (“erasers”). Demethylating enzymes are also specific for their substrates (mono-, di-, or tri-methylated residues) [29,34].

#### 2.2.3. Histone Phosphorylation

Histone phosphorylation is another major histone modification and is highly dynamic. Phosphorylation takes mostly place on serine (S) and threonine (Y) residues on histone tails and the balance of phosphorylation is established by phosphatases (“erasers”) and kinases (“writers”), which remove and add phosphate groups, respectively [29]. Various kinases and phosphatases have been described to actively contribute to histone phosphorylation thereby regulating cell functions including transcriptional regulation, apoptosis, cell cycle regulation, DNA repair and chromatin condensation [35]. Phosphorylation of the H2A variant H2AX is extensively been studied for its role in DNA damage. Upon DNA damage, phosphoinositide-3 kinases (ATM, ATR and PI-3K) phosphorylate H2AX on S139 (known as γ-H2AX). This histone mark is involved in DNA repair by recruiting DNA repair proteins to sites of lesions. The crosstalk with another phosphorylation event of H2AX (on Y142) mediates the decision between cell survival and apoptosis upon DNA damage. H2AX is in basal conditions phosphorylated on Y142 while phosphorylation of S139 is induced by DNA damage. If H2AX is not dephosphorylated on Y142 upon DNA damage, pathways involved in apoptosis will be initiated while dephosphorylation of Y142 allows recruitment of repair proteins by H2AX phosphorylated on S139 [36,37]. Phosphorylation of histone 3 has also been studied extensively with the most important example being phosphorylation of H3S10. AuroraB is the kinase responsible for phosphorylation of this residue in humans. H3S10 phosphorylation is linked with the cell cycle and increases across the whole genome during late G2-phase to initiate the condensation of chromosomes [35]. During the interphase, H3S10 phosphorylation is involved in transcriptional regulation and this in conjuction with H3K14 acetylation [35,38].

As mentioned above, histone modifications determine the global architecture of chromatin. This is illustrated by the existence of hetero- and euchromatin, each with their specific epigenetic marks. Heterochromatin contains permanently silenced genes and compacted regions such as centromers and telomeres. Typical heterochromatin marks include low levels of acetylation and methylation of H4K9, H3K27 and H4K20. Euchromatin is a less compacted region containing active genes. Genes that are actively transcribed contain high levels of acetylation, H3K4me3 and H3K36me3 [29,39].

### 2.3. Cooperation between Histone Modifications and DNA Methylation

A bidirectional cooperation between histone modifications and DNA methylation is important for the establishment of global epigenetic patterns as well as loci-specific gene regulation [40,41]. *De novo* global methylation during development has been suggested to be dependent on the pattern of H3K4 methylation, a positive mark for transcription. The pattern of H3K4 methylation is determined by the recruitment of HMTs by RNA polymerase II which is present in the majority of promoter associated CpG islands in the embryo’s genome. DNMT3a and DNMT3b are only recruited to chromatin regions that lack H3K4me, thereby methylating and repressing the remaining CpG sites such as those found in centromers and telomeres (heterochromatin). This pattern is maintained by DNMT1 upon replication [40]. The cooperation of histone modifications and DNA methylation on gene expression can be illustrated by the existence of at least three different chromatin states of gene promoters. A permissive (default) state is enriched by RNA polymerase II, histone acetylation and depleted of DNA methylation and H3K36me2. In order to become active, a specific stimulus is needed that provides additional regulatory proteins such as transcription factors. A bivalent state has been described in e.g., embryonic stem (ES) cells and is characterized by the presence of both active H3K4me2/3 (catalyzed by the Trithorax group) and repressive H3K27me3 (catalyzed by the polycomb repressive complex (PRC); consisting of EZH2, EED and SUZ12). This state is important for regulation of genes involved in differentiation and lineage commitment, e.g., genes that are kept inactive in ES, but become active upon differentiation or *vice versa*. At last, a repressed state exists in silenced genes and contains repressive marks like H3K27me3 and H3K9me2/3 [24,42,43]. DNA methylation act in concert with these repressive marks by a self reinforcing loop leading to permanent gene silencing. This loop is established by recruitment of repressor complexes that consist of HDACs, HMTs and DNMTs [2,40,41].

## 3. Epigenetic Changes in Multiple Myeloma

It is now widely accepted that upon genetic aberrations, epigenetic abnormalities are important for the initiation and progression of cancer, including MM [43,44]. Epigenetic aberrations in cancer have been described for most of the epigenetic mechanisms including DNA methylation and histone modifications and seem to be largely attributed to alterations in expression of histone modifying enzymes [17].

In cancer, global DNA hypomethylation of repetitive sequences (such as LINE-1 and Alu repeats), gene bodies and intergenic regions has been observed and contributes to genomic instability, transposon activation, proto-oncogene activation and loss of normal imprinting patterns. In addition, site-specific CpG hypermethylation of gene promoters of e.g., tumor suppressor genes results in gene silencing. This has been demonstrated for genes involved in cell cycle regulation, cell invasion, growth factor signaling, DNA repair, immune modulation and regulation of apoptosis [17,21]. In line with this, there is global hypomethylation of the LINE-1 and Alu repetitive elements in MM patients compared to normal control subjects [45]. Furthermore, global methylation levels of repetitive elements decreased upon disease progression from MGUS to MM and correlated with poor prognosis [46,47]. Interestingly, LINE-1 hypomethylation was associated with genomic aberrations such as translocations involving chromosome 14 and deletion of chromosome 13q [46], suggesting that hypomethylation of repetitive sequences can increase the vulnerability to genomic instability. Another study also demonstrated that the methylation patterns are linked to specific cytogenetic subgroups, including translocations and hyperdiploid state. The most distinct methylation pattern was associated with t(4;14) showing more frequent hypermethylation compared to other subgroups [47], underlining the poor prognosis associated with this translocation. Surprisingly, genome wide DNA methylation profiling revealed that in MM, hypomethylation of genes occurs early in MM pathogenesis and the degree increases upon progression while hypermethylation of genes seems to be a rare event [48]. Even though considered to be a rare event, hypermethylation of genes seems associated with the progression of MGUS to MM and to PCL [47]. In line with this, multiple studies using MM cell lines and primary MM samples have revealed loci-specific hypermethylation which is summarized in Table 1 and has been reviewed elsewhere [49]. Moreover, methylation of several of these genes was associated with poor prognosis of MM patients and include *SPARC*, *BNIP3* [50], *DAPK*, *RARβ* [51], *EGLN3* [52], *DLC-1* [53], *CDH-1* [54], *DCC*, *TGFβR2* [55], *CD9* [56] and *p16* [57]. One study even proposed that *TP73*, *p15*, *p16* and *ARF* methylation is an early event in MM pathogenesis because of hypermethylation in both MGUS and MM samples, while *SOCS-1* methylation was present in higher abundance in MM samples compared to MGUS and thus may be involved in the progression of MGUS to MM [58]. Furthermore, hypermethylation of the *RASD1* gene was associated with dexamethasone resistance [59]. These findings stress the importance of hypermethylated genes in MM. However, the molecular mechanisms underlying the poor prognosis associated with hypermethylation of certain genes remain largely undefined. Identifying these mechanisms will facilitate the implementation of DNA methylation in the clinic to predict prognosis and the response towards therapy. The mechanisms underlying the aberrant DNA methylation in MM are still unclear but are probably related to altered expression or activity of DNMT enzymes [49]. However, this cannot be the sole reason as DNMTs do not show sequence specificity and are dependent on accessory proteins to guide them to their targets. Alterations in accessory proteins or mutations in promoter sequences also may be responsible for aberrant DNA methylation.

**Table 1:** List of reported hypermethylated genes in multiple myeloma.

Biological function	Gene name (symbol)	Number of MM cells lines with methylation	Methylation frequency in primary MM samples	Poor prognosis	Reference
**regulation of apoptosis**	*XIAP-associated factor 1 (XAF-1)*	2	-		[60]
	*BCL2/adenovirus E1B 19 kDa protein-interacting protein 3 (BNIP3)*	3	5–13%	X	[50,51]
	*B-cell CLL/lymphoma 7C (BCL7c)*	3	21%		[50]
	*Growth Arrest and DNA Damage inducible γ (GADD45)*	1	19%		[50]
	*Death-associated protein kinase 1 (DAPK)*	1	5–20%	X	[51,54,57]
**regulation of cell cycle**	*Cyclin-dependent kinase inhibitor 2A (CDKN2A*, *p16)*	4	5–40%	X	[51,54,57,58,61]
	*Cyclin-dependent kinase 4 inhibitor B (CDKN2B*, *p15)*	-	5–30%		[51,54,57,58]
	*Cyclin-A1 (CCNA1)*	3	8%		[50]
**DNA repair**	*Methylated-DNA-protein-cysteine methyltransferase (MGMT)*	2	2–4%		[54,57]
**tumor suppression**	*Tumor protein 73 (TP73)*	2	12–45%		[54,57,58]
	*Tumor protein 53 (TP53)*	4	-		[62]
**signal transduction**	*Suppressor of cytokine signaling 1*, *3 (SOCS-1, -3)*	5	45–50%		[57,58,63,64]
	*Spleen tyrosine kinase (SYK)*	-	38%		[63]
	*Dexamethasone-induced Ras-related protein 1 (RASD1)*	5	8%		[59]
	*Deleted in liver cancer-1 (DLC-1)*	3	78%	X	[53]
	*Ras association domain-containing protein 1A (RASSF1A)*	-	2–15%		[54,58]
	*Stratifin (SFN)*	-	100%		[55]
**Wnt pathway**	*Wnt inhibitory factor 1 (WIF1)*	2	22%		[65]
	*Secreted frizzled-related protein 1, 2, 4, 5 (sFRP1, 2, 4, 5)*	4	4–50%		[65,66]
	*Dickkopf-related protein 1, 3 (DKK1, 3)*	2–4	16–32%		[65,67]
	*Adenomatous polyposis coli (APC)*	1	18%		[65]
**osteogenesis**	*Secreted protein acidic and rich in cysteine (SPARC)*	2	8%	X	[50]
**growth factor signaling**	*Transforming growth factor beta-receptor 2 (TGFβR2)*	-	45%	X	[55]
**hormone signaling**	*Estrogen Receptor (ESR1)*	-	15–80%		[51,55]
	*Retinoic acid receptor beta (RARβ)*	3	2–12%	X	[51,57]
	*Prostaglandin-endoperoxide synthase 2 (PTGS2)*	-	100%		[55]
**cell adhesion**	*Cadherin 1 (CDH1, E-cadherin)*	-	30–80%	X	[51,54,55]
	*Gap junction alpha-1 protein (GJA1)*	3	23%		[50]
	*CD9 antigen (CD9)*	2	-	X	[56]
	*A-kinase anchor protein 12 (AKAP12)*	2	13%		[50]
	*Deleted in colorectal carcinoma (DCC)*	-	45%	X	[55]
**coagulation**	*Tissue factor pathway inhibitor (TFPI2)*	2	10%		[50]
**hypoxia signaling**	*Egl nine homolog 3 (EGLN3)*	-	33%	X	[52]
**transcriptional repression**	*Hypermethylated in cancer 1 (HIC1)*	-	70%		[55]
**regulation of translation**	*Cytoplasmic polyadenylation element-binding protein 1 (CPEB1)*	4	50%		[50]
**transcription factor**	*Interferon regulatory factor 8 (IRF8)*	8	10%		[68]

Various mutations and translocations involving “writers”, “readers” and “erasers” of histone marks have been demonstrated to induce alterations in the pattern of histone modifications in cancer [69]. To our knowledge, little is known on aberrations of histone modifications in MM. So far, the best documented example in MM is the translocation t(4;14) which leads to overexpression of *MMSET* (NSD-2), a histone methyltransferase, in approximately 15% of the MM patients [70]. MM cells with t(4;14) show higher global levels of H3K36me2 and lower levels of H3K27me3 in comparison with non-t(4;14) MM cells [71]. Gene expression profiling revealed that *MMSET* regulates genes involved in the p53 pathway, apoptosis, cell cycle regulation, DNA repair and adhesion and knockdown of *MMSET* could negatively affect survival and adhesion of MM cells [71,72]. Genes upregulated by *MMSET* overexpression displayed H3K36me2 without the repressive H3K27me3 mark. In contrast, silenced genes were enriched for the repressive mark H3K27me3 and depleted of H3K36me2 [71]. Recent studies describe this phenomenon of functional antagonisms between these two histone marks as a consequence of an EZH2-MMSET axis [73,74]. In relation to gene silencing, we discovered a set of under-expressed genes enriched for H3K27me3 in MM patient samples compared to normal subjects [75]. Pharmacological intervention with the histone methylation inhibitor (HMTi) deazaneplanocin (DZnep) or the histone deacetylase inhibitor (HDACi) LBH589 led to re-expression of these silenced genes together with depletion of EZH2 and impaired survival of MM cells both *in vitro* and *in vivo* [75]. These results show that the MMSET-EZH2 axis can be an interesting target for therapy. Related to this, another member of the Polycomb group, Bmi-1, was shown to be upregulated in MM cells compared to normal plasma cells. On the functional level, Bmi-1 negatively regulates the expression of the pro-apoptotic gene Bim and thus functions as an oncogene. Knockdown of Bmi-1 decreased survival of MM cells by upregulation of Bim [76], what demonstrates its potential as a target for epigenetic therapy. In addition, chaetocin is a HMT inhibitor that has been evaluated in MM. Its anti-MM effects were ascribed to the induction of oxidative stress [77]. However, the HMT-inhibiting properties of chaetocin have not been thoroughly investigated in MM. Lastly, mutations in *WHSC1L1* (NSD-3), *MLL1-3* and in the histone demethylase *UTX* (KDM6A; removes H3K27me) have been described in MM patients [78,79]. The importance of these mutations in MM still needs to be resolved. To our knowledge, no reports on aberrant HDAC function in MM have been published. Nevertheless, HDAC inhibition in MM has been extensively studied, as described below.

## 4. Targeting Epigenetic Modifications in Multiple Myeloma

The above describes multiple epigenetic aberrations that have a major impact on MM cell biology and greatly influence development and progression of MM. Targeting epigenetic modifications therefore may represent a clinical useful alternative therapy for MM. Indeed, numerous studies have been conducted that address the potential of epigenetic modulating agents, in particular DNMTi and HDACi, in MM.

### 4.1. HDACi and Multiple Myeloma

Today, several HDACi have been developed that are being used in pre-clinical and clinical studies as anti-cancer agents (Table 2). HDACi can be classified in different groups according to their chemical structure: short chain fatty acids, hydroxamic acids, mercaptoketones, cyclic tetrapeptides and benzamides. On the functional level, most HDACi interfere with the Zn+ ion in the catalytic site of one of the HDAC classes I, II or IV or multiple classes (pan-HDACi). Given the fact that HDACs are responsible for deacetylation of proteins (histone and non-histone), inhibition of HDACs results in a wide variety of responses linked to epigenetic mechanisms and post-translational modifications of proteins [80,81,82]. The direct impact of HDAC inhibition on chromatin is the hyperacetylation of histones resulting in structural changes of the chromatin and alterations in gene expression. Gene expression profiling using MM cell lines revealed that upon HDAC inhibition, the expression of various genes could be up- or down-regulated [50,83,84,85,86,87]. Based on these studies, the predominant pathways relevant to MM that are affected by HDACi treatment include cell cycle regulation, apoptosis, cytokine signaling, adhesion and migration, proteasomal degradation, drug resistance and DNA damage. However, changes in gene expression are not the only explanation for the pleiotropic effects mediated by HDACi. Inhibition of HDACs affects also non-histone proteins regulated by acetylation. The true mechanism of HDACi is therefore likely to be a mixture of gene expression changes and deregulation of proteins through modulation of the acetylation status.

**Table 2:** List of commonly used HDAC inhibitors.

Chemical class	HDAC inhibitor	Reported targets
Benzamides	SNDX-275 (MS-275, Entinostat)	HDAC-1,-2,-3
	CI-994 (Tacedinaline)	HDAC-1, -2
	MGCD-0103	HDAC-1, -2, -3, 11
Short Chain Fatty Acids	Valproic acid (VPA)	Class I and IIa
	Sodium butyrate	Class I and IIa, IV
	Phenyl butyrate (S-HDAC-42, AR-42)	Class I, II
Cyclic Peptides	Depsipeptide (Romidepsin)	HDAC -1, -2
	Apicidin	Class I
Hydroxamic Acids	JNJ-26481585	Class I and II, IV
	Suberoylanilide hydroxamic acid (SAHA; Vorinostat)	Class I and II, IV
	Trichostatin-A (TSA)	Class I and II, IV
	LBH589 (Panobinostat)	Class I and II, IV
	ITF2357 (Gavinostat)	Class I and II
	PXD101 (Belinostat)	Class I and II, IV
	NVP-LAQ824 (Dacinostat)	Class I
	Suberoylanilide bis-hydroxamic acid (SBHA)	HDAC-1, -3
	RAS2410 (Resminostat)	HDAC-1, -3, -6
	ACY-1215 (Rocilinostat)	HDAC-6
	CR-2408	Class I, II, IV
Mercaptoketone	KD5170	Class I and II
Others	Tubacin	HDAC-6

**Figure 1:**
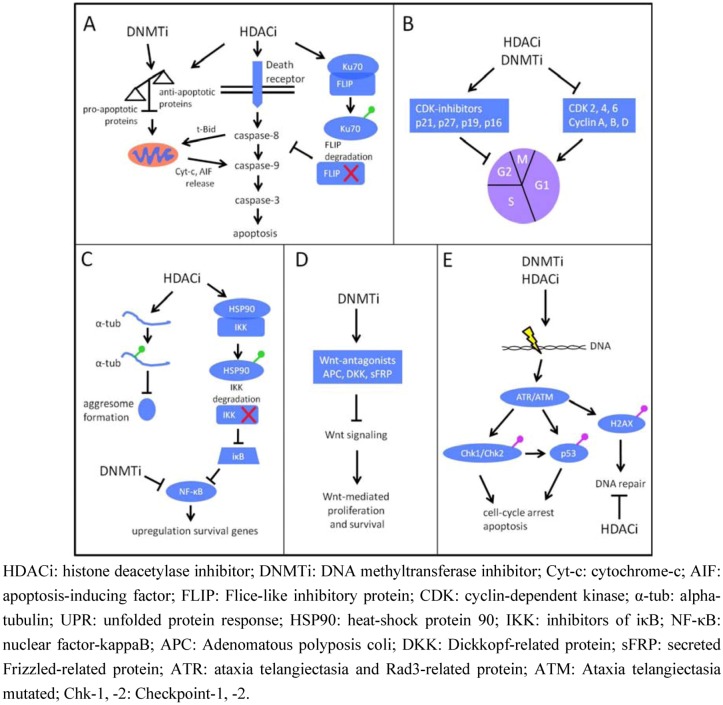
HDACi and DNMTi target the MM cell. (**A**) HDACi and DNMTi activate the intrinsic apoptotic pathway by disrupting the balance of anti- and pro-apoptotic molecules concomitant with the release of cytochrome-c and AIF from mitochondria. HDACi also activate the extrinsic pathway by inducing death-receptor expression. In addition, acetylation (green circle) of Ku70 by HDACi results in degradation of FLIP thereby relieving the inhibition of caspase-8 by FLIP. (**B**) HDAC and DNMTi induce cell cycle arrest by inducing CDK inhibitors and repressing CDK and cyclin proteins. (**C**) HDACi leads to hyperacetylated alpha-tubulin. Thereby, the formation of aggresomes is inhibited what leads to attenuation of the UPR. HDACi also induce acetylation of HSP90 resulting in IKK degradation and inhibition of NF-κB. DNMTi also inhibit NF-κB activity. Both pathways form the rationale for combination therapy with bortezomib. (**D**) DNMTi induce the expression of Wnt-antagonist resulting in abrogation of Wnt-mediated proliferation and migration in advanced stages of MM. (**E**) HDACi and DNMTi can act as DNA damaging agents by activating ATR/ATM and inducing phosphorylation (purple circle) of Ckh-1, -2, p53 and H2AX. This results in cell cycle arrest and apoptosis. In addition, HDACi can inhibit DNA repair mechanisms.

The pro-apoptotic and cell cycle arresting properties are the best characterized features of HDACi and have been extensively reviewed by our group and others [44,88]. HDACi can activate both the intrinsic and extrinsic apoptotic pathway associated with caspase cleavage. HDACi-mediated activation of the intrinsic pathway can be achieved by: (I) upregulation of pro-apoptotic or downregulation of anti-apoptotic Bcl-2 family members, (II) downregulation of inhibitors of apoptosis (IAP) and (III) mitochondrial changes including release of apoptosis inducing factor (AIF) and cytochrome-c into the cytosol (Figure 1A) [83,84,89,90,91,92,93,94]. For example, LBH589 and SBHA were able to induce the expression of pro-apoptotic Bim *in vitro* [94,95]. However, the induction of pro-apoptotic molecules on itself may not be enough for efficient induction of apoptosis as these pro-apoptotic molecules can be sequestered by anti-apoptotic Bcl-2 and Bcl-xL. Thus, targeting these interactions with Bcl-2 mimetics could overcome this problem. This has been illustrated in a study by Chen *et al*. where ABT-737, a Bcl-2 mimetic, potentiated SBHA-mediated cell death by releasing Bim from Bcl-2 and Bcl-xL complexes [95]. Upregulation of death-receptors and -ligands, caspase-8 cleavage and downregulation of Flice-like inhibitory protein (FLIP; caspase-8 inhibitor) are part of the mechanisms by which HDACi activate the extrinsic pathway (Figure 1A) [85,93,96]. Interestingly, FLIP is stabilized by interaction with Ku70, a protein that has been shown to be regulated by acetylation. Hyperacetylation of Ku70 by HDACi disrupts the interaction with FLIP resulting in FLIP degradation and loss of inhibition of caspase-8 *in vitro* [97]. This provides an additional mechanism to activate the extrinsic pathway (Figure 1A). TNF-related apoptosis-inducing ligand (TRAIL) induces apoptosis by activating death-receptors. Since HDACi can activate the extrinsic apoptotic pathway, MM cells could be sensitized to the effects of TRAIL by the use of HDACi *in vitro* [90,96]. In addition, combination studies with conventional chemotherapeutic agents such as dexamethasone, doxorubicin and melphalan showed *in vitro* synergistic activity with several HDACi, even in cell lines resistant to these agents [84,85,91,92,98,99,100,101]. In an *in vivo* xenograft MM model, the combination of LBH589 and melphalan significantly reduced tumor load and serum M-spike levels compared to single agent treatment [101]. Altogether, these studies reveal how HDAC inhibition directly activates apoptotic programs and form the rationale for combination therapies with cytotoxic agents.

The apoptosis observed by HDAC inhibition is often associated with a G0/G1-phase arrest. In MM cell lines and patient samples, HDACi induced a G0/G1-phase arrest what has often been linked to the upregulation of the cyclin-dependent-kinase (CDK)-inhibitor p21 by p53-dependent and -independent ways (Figure 1B) [83,84,96,98,102]. Other effects that may contribute to the arrest is the upregulation of other CDK-inhibitors like p27 [91,103,104] and p19 [105] and/or the decrease of cyclin and CDK family members (Figure 1B) [83,84,89,93,105]. Of note, it is not yet clear whether the observed changes in expression of cell cycle regulatory proteins are a consequence of altered transcription rather than alterations in protein stability or degradation. More detailed studies could further explain these observations and highlight underlining mechanisms of cell cycle deregulation.

Among the pleiotropic effects of HDACi, the proteasome system is also affected as evidenced by decreased activity of the 20S proteasome activity in MM cells [85,98]. HDACi were also shown to downregulate members of the proteasome pathway, including genes encoding different subunits of the 26S proteasome and ubiquitin conjugating enzymes *in vitro* [85]. Furthermore, HDAC inhibition enhanced the cytotoxic effects of bortezomib both *in vitro* and *in vivo* [84,85,90,104,106,107,108]. The mechanism of synergy between HDACi and bortezomib is multifactorial. The main mechanism is the disruption of protein degradation following inhibition of the proteasome and aggresome pathway by bortezomib and HDACi, respectively. Inhibition of the proteasome by bortezomib results in the activation of the unfolded protein response (UPR) and aggresome formation to cope with the accumulation of proteins that need to be degraded. The UPR response is dependent on HDAC6 that deacetylates alpha-tubulin (α-tub), thereby allowing migration of protein aggregates on tubulin strands and aggresome formation [109]. LBH589 treatment leads to hyperacetylated α-tub (ac-tub), thereby disrupting the normal UPR and enhancing the effect of bortezomib (Figure 1C) [106]. Furthermore, ac-tub and downstream ER stress markers such as splicing of X-box binding protein 1 (XBP-1), upregulation of IRE1 and protein kinase RNA-like endoplasmic reticulum kinase (PERK) and phosphorylation of eukaryotic initiating factor (eIF2) have been demonstrated using ACY-1215, a specific HDAC6 inhibitor [108]. One additional mechanism involves the link between HDACi, bortezomib and the nuclear factor-kappaB (NF-κB) pathway. NF-κB stimulates survival and progression of the MM tumor [109,110]. NF-κB activation is dependent on the proteasomal degradation of its inhibitor IκB. The degradation of IκB is inhibited by bortezomib, resulting in decreased NF-κB activation. On the other hand, inhibition of HDACs results in the downregulation of NF-κB and the reduction of the NF-κB DNA binding capacity [98,105,109]. Furthermore, HDAC6 inhibition leads to acetylation of heat shock protein 90 (HSP90), what disrupts its interaction with inhibitors of IκB (IKK). This results in degradation of IKK and subsequently inhibition of the degradation of IκB (Figure 1C) [111]. These studies formed the rationale for implementing the combination of bortezomib and HDACi in clinical trials as discussed below.

Very recently, there has been a growing interest in the link between HDACi and DNA damage (Figure 1E) [112]. Some evidence supports this link as well in MM. The HDACi PDX-101 and KD-5170 have been shown to induce DNA damage as evidenced by phosphorylation of H2AX on ser139 (γ-H2AX). This was associated with p53 phosphorylation and oxidative stress as shown by p38 mitogen-activated protein kinase and c-Jun *N*-terminal kinase activation [90,113]. Another HDACi, SDNX-275, could enhance the DNA damage response induced by the alkylating agent melphalan in MM cell lines. On the molecular level, phosphorylation of H2AX on ser139 (γ-H2AX) was increased together with p53 phosphorylation and checkpoint activation as evidenced by Chk-1 and -2 phosphorylation [100]. HDACi also influenced the formation of RAD51 foci that are involved in repair of DNA by homologous recombination. By this action, MM cells could be sensitized to the effect of ionizing radiation [114]. The above results clearly show an induction of the DNA damage response upon HDAC exposure. It remains to be defined what kind of damage is elicited and whether this response is also relevant *in vivo*.

**Figure 2:**
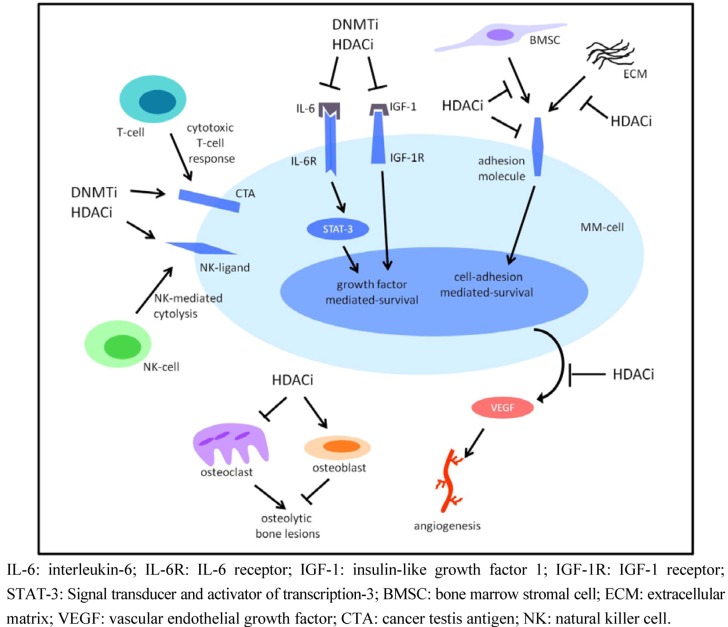
HDACi and DNMTi target the BM microenvironment. HDACi and DNMTi affected several pathways in relation to the BM-microenvironment. Firstly, HDACi and DNMTi inhibit cytokine signaling by reducing expression of IGF-1R, IL-6R and IGF-1. In addition, HDACi decrease expression of adhesion molecules, thereby attenuating adhesion-mediated survival. HDACi also inhibit the expression and secretion of VEGF, thereby decreasing angiogenesis. Both HDACi and DNMTi upregulate CTAs and NK-ligands. This leads to an increased potential of cytotoxic T-cell responses and NK-mediated cytolysis. HDACi are furthermore shown to reduce osteoclast numbers and stimulate osteoblast generation, thereby inhibiting the development of osteolytic bone lesions.

In addition to targeting the MM cells itself, HDACi also affect the BM microenvironment. Microarray studies showed that HDACi downregulated the expression of genes involved in cytokine signaling such as IGF-1, IGF-1 receptor (IGF-1R), and IL-6R (Figure 2) [85,87]. IGF-1 is a major growth factor for MM cells produced by the BM microenvironment and could potentially protect MM cells from HDACi-mediated cell death *in vivo* [1]. One study showed that exogenous IGF-1 attenuated SAHA-induced cell death in MM-1S cells [85], while other studies showed no effect of exogenous IGF-1 on apoptosis mediated by HDACi [89,108]. This discrepancy could be explained by differences in cell lines or HDACi used. We showed that IGF-1 is involved in MM cell survival by epigenetic silencing of pro-apoptotic Bim. Treatment with LBH589 could overcome this silencing by inducing H3K9ac and reducing H3K9me2 in the Bim promoter [94]. Furthermore, combination therapy of LBH589 with an IGF-1R inhibitor, picropodophyllin (PPP), had synergistic effects on MM cell survival *in vitro*. In the syngeneic murine 5T33MM model, this combination decreased BM tumor load and increased survivalcompared to single agent treatment [83], demonstrating the *in vivo* relevance. The role of IL-6, another major growth factor for MM, in HDACi-mediated cell death has also been explored. The use of exogenous IL-6 was unable to overcome cell death by HDACi [89,93,98,108]. Signal transducer and activator of transcription-3 (STAT-3) is a transcription factor activated by IL-6 signaling and is often constitutive activated in MM cells. STAT-3 gene targets include anti-apoptotic Bcl-2 family members such as Bcl-xL and Mcl-1 [115]. Constitutive and induced phosphorylation of STAT-3 was abrogated upon HDAC inhibition, indicating the involvement of STAT-3 in the downregulation of anti-apoptotic proteins [87,93]. As mentioned earlier, contact of MM cells with BMSC and the extracellular matrix (ECM) results in the upregulation of growth factors by both cell types [1]. The use of HDACi can downregulate CD138 and thus abrogate CD138-mediated survival mechanisms (Figure 2) [85]. In addition, co-culture with BMSC could not overcome HDACi-mediated anti-proliferative and apoptotic effects demonstrating that HDACi can circumvent adhesion-mediated survival mechanisms [92,98,108,113]. In summary, HDAC inhibition attenuates crucial survival pathways related to the BM microenvironment. This is an important advantage of HDACi as these pathways could limit the use of HDACi in the clinic. The idea that HDACi can partially overcome the protective effects of the BM microenvironment is further strengthened by the use of xenograft and syngeneic MM models in which HDACi could attenuate MM progression and increase survival of MM inoculated mice [86,90,98,103,107]. A major consequence of MM disease is the development of osteolytic lesions. *In vitro*, HDACi attenuated osteoclast formation alone [116] and in combination with bortezomib (Figure 2) [113]. Furthermore, in the syngeneic murine 5T2MM model, we demonstrated that JNJ-26481585 decreased the amount of osteoclasts [103]. This effect was even enhanced in combination with bortezomib. The combination of JNJ-26481585 and bortezomib also stimulated osteoblast generation [107]. 

MM is also characterized by increased BM angiogenesis. The use of valproic acid (VPA) reduced the secretion of VEGF by MM cell lines (Figure 2) [117]. In addition, the formation of new blood vessels *in vitro* was abrogated by VPA [116]. Using the 5T33 and 5T2MM model, we also confirmed the anti-angiogenic properties of HDACi *in vivo* [103]. At last, HDACi can also modulate the immune response towards MM cells. Our group showed that LBH589 upregulated CD9 expression in MM cells, thereby making the cells more vulnerable for natural killer cell (NK)-mediated cytolysis (Figure 1H) [56]. Another study could demonstrate that VPA upregulated ligands for NK recognition in MM cells and subsequently increased cytolysis by NK-cells (Figure 2) [118]. These data suggest that HDACi can be useful together with the use of immunomodulatory drugs or vaccination strategies.

### 4.2. DNMTi and Multiple Myeloma

Aberrant DNA methylation can be targeted with the use of DNMTi leading to demethylation of DNA. Commonly used DNMTi are the cytidine analogs 5-azacytidine (AZA) and 5-aza-2'-deoxycytidine (decitabine; DAC). Their anti-tumor effects have been linked to two mechanisms: passive DNA demethylation and induction of DNA damage. DAC and AZA (though with less specificity) are incorporated in the DNA where they covalently trap DNMT enzymes resulting in DNMT degradation and passive DNA demethylation. However, DAC and AZA also appear to be cytotoxic as a DNA damage response can be elicited upon treatment [119]. There are only limited studies that address the anti-MM effects of AZA or DAC and most of them focused mainly on the demethylation properties of DNMTi. In MM cell lines, DAC has been shown to restore the expression of p16 by DNA demethylation. In addition, DAC could induce a G0/G1- and G2/M-phase arrest linked with p21 or p38, respectively (Figure 1B) [120]. Zebularine, another cytidine analog, has been shown to demethylate and re-express the p53 gene in MM cell lines. Following combination with a p53 activating peptide, significant apoptosis could be observed in cell lines originally containing hypermethylation of the p53 gene [62]. Another study could demonstrate the anti-MM effects of AZA both *in vitro* and *in vivo*. This was associated with p16 re-expression, G0/G1-phase arrest and cleavage of PARP and caspase proteins (Figure 1A,B). Also genes involved in apoptotic pathways are targeted by AZA. XIAP-associated factor 1 (XAF-1) inhibits IAP proteins, thereby preventing inhibition of caspase proteins by IAPs. XAF-1 has been shown to be hypermethylated in MM cells lines and could be re-expressed by AZA treatment (Figure 1A). In addition, AZA synergistically induced apoptosis with arsenic trioxide *in vitro* and compared to single agent treatment the combination significantly prolonged survival in a xenograft MM model [60]. Furthermore, critical survival pathways for MM cells are also targeted by DNMTi. For example, AZA could suppress the IL-6 and NF-κB pathway (Figure 1C, Figure 2) [61]. Synergism has been demonstrated with other agents targeting the NF-κB pathway such as the specific HSP90 inhibitor NVP-HSP990 [121]. The Wnt-pathway is implicated in MM pathogenesis affecting proliferation and migration [122]. In advanced stages of MM, Wnt-antagonists encoding genes such as APC, DKK and sFRP are hypermethylated which is consistent with the constitutive activation of the Wnt-pathway. Using DAC, these antagonists can be re-expressed and attenuate Wnt-signaling in MM cells (Figure 1D) [65,67]. Besides the candidate-gene approach, genome-wide gene expression studies have been performed in MM cells using DAC [50]. Very recently, a method has been developed to predict the response of DAC *in vitro*. This method is based on a DNA methylation score (DM score) that is related to the expression of methylation-regulated genes. A high DM score was found to correlate with poor patient survival and high sensitivity of MM cell lines and primary MM samples to DAC [123]. Whether this approach will be useful to predict patients’ responses *in vivo* is not sure as a direct link between DNA methylation changes, differences in gene expression and clinical effects has not been shown [124,125]. As mentioned above, DNMTi are also described to induce a DNA damage response (Figure 1E). However, studies addressing this aspect in MM are limited. Kiziltepe *et al*. demonstrated that AZA showed a marked increase in H2AX, Chk-2 and p53 phosphorylation in MM cell lines and observed synergistic interactions when AZA was combined with doxorubicin and bortezomib [126]. In conclusion, DNMTi have significant *in vitro* anti-MM activity. The mechanisms involve not only changes in gene expression but also induction of DNA damage. Therefore, the induction of a DNA damage response after DNMTi treatment warrants further investigation to find out the exact contribution of this response in the anti-tumor effects of DNMTi.

### 4.3. Combining HDACi and DNMTi in Multiple Myeloma

DNA methylation and histone modifications act together to regulate gene expression [41]. Consequently, targeting both DNA methylation and histone acetylation simultaneously provides another strategy to target epigenetic aberrations in cancer [17]. In MM, there have been a few studies conducted to investigate the effects of the combination of a DNMTi and HDACi. TSA and DAC alone and in combination have been shown to induce genome wide changes in the expression of genes relevant to MM pathogenesis. There were genes regulated by TSA or DAC alone but also a significant subset of genes affected by the combination of both agents [50]. In MM cell lines, DAC and sodium-butyrate have synergistic effects on MM cell growth and apoptosis. This was associated with a synergistic interaction between DAC and sodium-butyrate on p16 re-expression [127]. Interestingly, phenylhexyl isothiocyanate (PHI) could act both as a HDACi and hypomethylating agent. PHI induced p16 DNA hypomethylation together with histone H3 hyperacetylation in MM cells. Furthermore, MM cells were subjected to G0/G1-phase arrest and apoptosis associated with p21 induction and disruption of the mitochondrial membrane. Also the expression of the IL-6R was reduced, demonstrating that multiple mechanisms contribute to PHI-mediated cell death (Figure 1A,B, Figure 2) [128]. An interesting link with the immune system has also been shown. Cancer testis antigens (CTA) like melanoma-associated antigen (MAGE) are involved in CD8+ cytotoxic T-lymphocyte (CTL) responses towards tumor cells. The combination of VPA and AZA induced the expression of MAGE-A1 in myeloma cells. Consequently, a MAGE-A1 specific CTL response was elicited upon treatment of the MM cells with VPA and AZA [129]. A similar study observed MAGE-A3 induction in MM cells upon treatment with AZA and the HDACi MGCD-0103, followed by MAGE-A3 specific CTL responses [130]. These studies highlight the potential of using HDACi and DNMTi in order to augment a tumor cell specific CTL responses *in vivo* (Figure 2).

## 5. HDACi and DNMTi in the Clinic

Several HDACi are implemented in clinical trials, either as monotherapy or in combination with conventional agents for the treatment of MM. LBH589 (panobinostat), SAHA (vorinostat), PXD101 (belinostat), depsipeptide (romidepsin) and ITF2357 (gavinostat) have been tested as monotherapy for relapsed/refractory MM (Table 3). The most common side effects were diarrhea, nausea, thrombocytopenia, neutropenia and renal dysfunction mostly of grade 2. In addition, also some patients developed adverse effects of grade 3 or higher. However, the clinical activity was modest as only a minority of patients showed a minimal response or disease stabilization (Table 3) [131,132,133,134,135]. Based on this, HDACi are now being tested in combination therapy with conventional agents (see Table 3 for more details) [136,137,138]. Phase I clinical studies using vorinostat in combination with bortezomib showed favorable tolerance and a good overall response in bortezomib-refractory patients [139,140,141]. Vorinostat has also been evaluated in a phase I study together with the classic lenalidomide, bortezomib and dexamethasone combination in newly diagnosed patients. Grade 3 adverse effects were observed in 10% of the patients. Nevertheless, the overall response rate was 100% [142]. Combination of vorinostat with lenalidomide/thalidomide and dexamethasone in refractory patients showed a response rate of 73% with favorable tolerance in a phase I/II trial [143]. Panobinostat has also been evaluated in a phase II trial in combination with bortezomib and/or dexamethasone and demonstrated good tolerance and an overall response rate of 35% [144]. First results of the combination of panobinostat and carfilzomib in phase I studies has shown good tolerability and 35% overall response rate [145,146], which seems similar to the combination with bortezomib. Also the combination of romidepsin and bortezomib or dexamethasone has been evaluated in a phase I/II clinical trial. However, 60% of patients showed grade 3 adverse effects of which thrombocytopenia and fatigue were the most common. Nevertheless, the overall response rate was 72% [147]. Based on the promising results of phase I/II trials, two large multicenter phase IIb/III studies are currently further evaluating the effect of vorinostat in combination with bortezomib in refractory patients [148,149]. The combination of panobinostat and bortezomib and/or dexamethasone is also under evaluation in a large multicenter phase III clinical trial in refractory patients [150]. The above phase II/III studies are mostly focusing on refractory patients. The combination of HDACi and bortezomib is also being evaluated in phase II/III clinical trials in newly diagnosed patients [136]. Furthermore, clinical trials have been initiated to evaluate the immunomodulatory effect of HDACi in a transplantion setting [151]. A phase I study of the combination of vorinostat and lenalidomide demonstrated no severe side effects and an improvement of the graft in 4 out of 16 patients [152]. Overall, the use of HDACi in the clinic is most promising when combined with other agents. To cope with the side effects, optimal dose scheduling and treatment regimes are being evaluated.

**Table 3:** Overview of clinical trials with epigenetic modulating agents in MM.

Drug	Drug	Combination with	Myeloma patients	Response	Reference
**Vorinostat**	I	-	relapsed/refractory (n = 13)	1 MR	[134]
				9 SD	
**Belinostat**	I	-	relapsed/refractory (n = 4)	1 SD	[135]
**Panobinostat**	Ia/II	-	relapsed/refractory (n = 12)	1 PR	[131]
**Romidepsin**	II	-	relapsed/refractory (n = 13)	4 SD	[132]
**Gavinostat**	II	-	relapsed/refractory (n = 19)	6 SD	[133]
**Vorinostat**	I	Bortezomib	relapsed/refractory (n = 23)	2 VGPR	[141]
				13 PR	
				10 SD	
**Vorinostat**	I	Bortezomib	relapsed/refractory (n = 6)	1 VGPR	[140]
				4 MR	
				1 SD	
**Vorinostat**	I	Bortezomib	relapsed/refractory (n = 34)	9 PR	[139]
				2 MR	
				20 SD	
**Vorinostat**	I	Lenalidomide	newly diagnosed (n = 30)	10 CR	[142]
		Bortezomib Dexamethasone		15 VGPR	
**Vorinostat**	I/II	Lenalidomide Bortezomib Dexamethasone	relapsed/refractory (n = 64)	8 CR	[143]
				4 VGPR	
				22 PR	
				9 MR	
				9 SD	
**Panobinostat**	II	Bortezomib Dexamethasone	relapsed/refractory (n = 55)	1 CR	[144]
				18 PR	
				10 MR	
				20 SD	
**Panobinostat**	I	Carfilzomib	relapsed/refractory (n = 17)	2 VGPR	[146]
				6 PR	
				1 MR	
**Panobinostat**	I/II	Carfilzomib	relapsed/refractory (n = 10)	ongoing	[145]
**Romidepsin**	I/II	Dexamethasone Bortezomib	previously treated (n = 25)	2 CR	[147]
				13 PR	
				3 MR	
				2 SD	
**Romidepsin**	I/II	Bortezomib	relapsed/refractory (recruiting)	ongoing	[136]
**Vorinostat**	IIb	Bortezomib	relapsed/refractory (n = 143)	ongoing	[149]
**Vorinostat**	III	Bortezomib	relapsed/refractory (n = 637)	ongoing	[148]
**Panobinostat**	III	Bortezomib	relapsed/refractory (n = 672)	ongoing	[150]
**Panobinostat**	I/II	Lenalidomide Bortezomib Dexamethasone	newly diagnosed (recruiting)	ongoing	[136]
**Vorinostat**	III	Lenalidomide	newly diagnosed (recruiting)	ongoing	[136]
		Thalidomide			
		Bortezomib			
**Vorinostat**	I	Lenalidomide	post transplant (n = 16)	4 improved responses	[152]
**Azacytidine**	II	Lenalidomide	partial remission or plateau (n = 14)	6 CTA upregulation	[153]
				3 CTL responses	
**Azacytidine**	I	Lenalidomide	Transplantation eligible	ongoing	[136]
			(recruiting)		
**Azacytidine**	I/II	Lenalidomide	relapsed/refractory	ongoing	[136]
		Dexamethasone	(recruiting)		
**Decitabine**	I	-	relapsed/refractory	ongoing	[136]

CR: complete response; VGPR: very good partial response; PR: partial response; MR: minimal response; SD: stable disease; CTA: cancer testis antigen; CTL: CD8^+^ cytotoxic T-lymphocyte.

At this moment, DNMTi are considerably less used in the clinic for treatment of MM compared to HDACi. Some clinical trials are ongoing to evaluate the safety of DNMTi as monotherapy or in combination with lenalidomide or dexamethasone in MM (Table 3) [136]. In addition, the potential combination of hypomethylating agents with immunomodulatory drugs comes from the pre-clinical proof that AZA induced CTA-specific CTL reactions in MM [129,130]. This route has been evaluated in a phase II clinical trial in MM patients. Patients were treated with a combination of AZA and lenalidomide before and during autologous lymphocyte collection. After stem cell transplantation, autologous lymphocyte infusions were performed in the patients. AZA and lenalidomide treatment resulted in upregulation of several CTAs in the MM cells. As a consequence, CTA-specific CTL reactions targeting the MM cells were observed demonstrating that an adaptive immune response is induced by AZA [153]. However, it is clear that additional clinical trials are needed before any definite conclusion can be drawn on the use of DNMTi in the clinic as anti-MM agents.

## 6. Conclusions and Perspectives

Epigenetic aberrations have now been recognized to contribute to the development and progression of cancer, including MM. Specific epigenetic alterations are rapidly being discovered and underline the altered epigenetic state in cancer. These alterations could be useful as prognostic markers for MM patients. Currently, DNA methylation is the most promising to use as prognostic marker. First, DNA methylation is relatively easy to investigate. Furthermore, gene specific DNA hypermethylation is often associated with poor prognosis proving its potential as a prognostic marker. Elucidating why some genes are linked with poor prognosis will further facilitate the use of DNA methylation for patients’ risk stratification. Lastly, global DNA hypomethylation is linked with genomic instability, which is an important feature of MM development and progression. Further investigations are needed to elucidate what molecular mechanisms are involved in this DNA hypomethylation-correlated genomic instability. For example, differences in hypomethylation of genomic regions in the proximity of common MM-associated translocation breakpoints or deletions between MGUS, MM and PCL patients can reveal important drivers of MM progression. In addition, the poor prognosis associated with the t(4;14) translocation involves the overexpression of the histone methyltransferase MMSET. This demonstrates that aberrant histone methylation is related to poor prognosis and may even act in concert with abnormal DNA methylation.

To reverse the altered epigenetic state of cancer cells, epigenetic modulating agents have been developed and evaluated in numerous pre-clinical studies. Today, a number of clinical trials have been conducted or are ongoing to evaluate their potential as monotherapy or in combination with conventional agents for the treatment of MM. Although hopeful results have been obtained, especially in a combination setup, the observed responses are still modest and the side-effects are often quite severe, probably as a consequence of the pleiotropic effects of these agents. Thus, although the use of epigenetic modulating agents in cancer therapy is a promising area, there are still important challenges that need to be resolved. Firstly, most of the pre-clinical studies, demonstrating the efficient targeting of both cancer cells and their interactions with the environmental cells by multiple mechanisms, have been conducted *in vitro*. However, *in vivo* evidence of these *in vitro* identified anti-MM mechanisms is mostly lacking. A better understanding of these mechanisms in an *in vivo* context is necessary to elucidate relevant and dominant mechanisms of action. Another important challenge is to identify suitable biomarkers for the response towards epigenetic modulating agents. Due to a lack of suitable biomarkers, today it is very difficult to monitor the biological effect of these agents and thus their clinical activity. Yet another major challenge is the high toxicity profile of these agents. In order to lower side effects, suboptimal treatment schedules have been designed. However, this may very well be an explanation as to why these agents fail to induce an anti-MM effect. Lastly, an essential challenge for the clinic is to develop ways to predict the response of patients towards epigenetic agents.

To address the above challenges, more detailed pre-clinical studies need to be performed. Firstly, better understanding of the mechanisms *in vivo* will lead to the identification of novel, more specific targets that are related to the activity of epigenetic modulating agents. These targets can then be used for the development of more specific drugs in order to reduce the side-effects associated with the broad spectrum epigenetic drugs. Today, most of the studies have mainly focused on DNMTi and HDACi. There is now increasing interest to selectively target histone methylation, but so far only a few studies addressed this potential in MM. Identification of the relevant targets will also open the possibility to design other combination setups besides those with conventional agents. For example, thorough understanding of the DNA damage response upon treatment with epigenetic modulating agents can provide the rationale for the combination with specific DNA repair inhibitors. Secondly, these in-depth mechanistic studies will lead to the identification of biomarkers to follow the response to epigenetic modulating agents. This will be useful to optimize the therapeutic window and dose scheduling of these agents and thus reduce the side-effects. Thirdly, identification of those patients that would really benefit from epigenetic therapy can lead to better designs of clinical trials. So far, predictions to the *in vitro* response towards DNMTi have been achieved using differences in expression of methylation regulated genes after DNMTi treatment. However, prediction towards HDACi has not been demonstrated so far. One way to predict responses might be the identification of shared epigenetic aberrations in patients’ groups that can specifically be targeted by broad spectrum or more specific epigenetic agents. At last, translocations and mutations of genes involved in histone methylation are also present in MM. Therefore, studies are needed to address the role of aberrant histone methylation to identify suitable targets for drug development. Preliminary data point out to the potential of the MMSET-EZH2 axis and Bmi-1 as such targets. 

In conclusion, in MM epigenetics has grown out to an essential research area where important challenges are yet to be resolved and interesting possibilities will be uncovered the next few years through further investigations.
